# Using Faecal Cortisol Metabolites to Assess Adrenocortical Activity in Wild‐Living Alpine Marmot *Marmota marmota* : A Biological Validation Experiment

**DOI:** 10.1002/ece3.70662

**Published:** 2025-01-29

**Authors:** Friederike Zenth, Elena Morocutti, Rupert Palme, Sandro Nicoloso, Stefano Giacomelli, Sabine Macho‐Maschler, Ilse Storch, Luca Corlatti

**Affiliations:** ^1^ Chair of Wildlife Ecology and Management University of Freiburg Freiburg im Breisgau Germany; ^2^ Department of Life Sciences University of Siena Siena Italy; ^3^ Stelvio National Park Bormio Italy; ^4^ Department of Biological Sciences and Pathobiology, Experimental Endocrinology University of Veterinary Medicine Vienna Vienna Austria; ^5^ Research, Ecology and Environment Dimensions (D.R.E.Am. Italia) Pistoia Italy; ^6^ Veterinary Service, DFTVCS ATS Della Montagna Sondrio Italy

**Keywords:** capture, FCMs, marmot, stress, validation, welfare

## Abstract

Faecal cortisol/corticosterone metabolites (FCMs) have become increasingly popular as an easy‐to‐sample, non‐invasive and feedback‐free alternative to assess glucocorticoid (GC) levels, key components of the neuroendocrine stress response and other physiological processes. While FCMs can be a powerful aid, for instance, for gaining insights into ecological and evolutionary processes, as well as to assess animal welfare or impacts of anthropogenic stressors on wildlife populations, this method comes with specific challenges. Because GCs are heavily metabolised before excretion, it is critical to validate the enzyme immunoassays (EIAs) used to measure FCMs. Additionally, because species may differ in metabolite profiles, assay validation must be performed separately for each focal species. Despite this, the use of unvalidated assays remains widespread. We performed a biological validation experiment to test a set of EIAs to measure FCMs and adrenocortical activity in free‐living Alpine marmots *Marmot marmota*. We capitalised on capture and handling as part of a relocation project of marmots under the assumption that capture, and handling represent a stressful event and tracked changes in FCM levels over the following 48 h. Faeces samples collected at capture were assumed to return baseline FCM levels. Of the three EIAs tested, only the 11‐oxoetiocholanolone ‘72T’ EIA detected an increase in FCM levels about 18 h after capture. This result paves the way for future studies using FCMs to investigate the adrenocortical activity in this species.

## Graphical abstract

Faecal cortisol/corticosterone metabolites (FCMs) are a powerful, non‐invasive and feedback free aid to assess adrenocortical activity and stress in wildlife, yet this methods comes with challenges: Because glucocorticoids are heavily metabolised prior to excretion and metabolite profiles are species specific, validation of assays used to assess FCMs is mandatory. We validate an enzyme immunoassay (EIA), the 11‐oxoetiocholanolone ‘72T’ EIA, though a biological validation experiment in the protected Alpine marmot, thus paving the way for future studies using FCMs to investigate adrenocortical activity in this species.

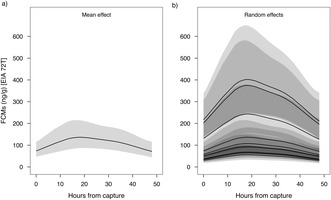

## 1 Introduction

Growing interest in understanding and predicting wildlife responses to environmental changes and human‐induced stressors has led to extensive research on stress indicators in various species (Baker, Gobush, and Vynne 2013). Stress can be defined as conditions in which environmental challenges surpass an organism's natural regulatory capacities, often characterised by unpredictability and/or a lack of control (Koolhaas et al. 2011). Stress responses can manifest in various forms, including behavioural changes such as increased vigilance and altered activity patterns (Tarjuelo et al. 2015), as well as physiological responses, with glucocorticoid (GC) levels serving as a key indicator of the endocrine stress response (Sheriff et al. 2011).

Adrenal glucocorticoids play a broad role in energy regulation, homoeostasis and other physiological aspects, including the physiological stress response (Jenni‐Eiermann et al. 2015; Sapolsky, Romero, and Munck 2000). The perception of a stressful situation triggers the sympathetic‐adrenomedullary system and the hypothalamic–pituitary‐adrenocortical (HPA) axis (Von Holst 1998). When the HPA axis is activated, it prompts the release of glucocorticoids (i.e., corticosterone [birds, amphibians, reptiles and some rodents] or cortisol [fish and most mammals]), which help an individual to mobilise energy (Von Holst 1998; Romero and Butler 2007). While adrenocortical activity is an adaptive response to meet metabolic and physiological requirements needed for behavioural activity (Koolhaas et al. 2011), chronic activation, for example due to prolonged exposure to unpredictable food shortages or anthropogenic disturbances (Mateo and Cavigelli 2005; Grundei et al. 2024), have been linked to negative effects (Mateo and Cavigelli 2005) such as immunosuppression, physiological disorders (Romero and Butler 2007) or changes in behaviours (Sapolsky, Romero, and Munck 2000), ultimately affecting key life history traits (Wahl et al. 2011).

GCs (and their metabolites) can be measured by enzyme immunoassays (EIAs) in several biological matrices (i.e., blood, faeces, hair, feathers and tissues [Palme 2005, 2019]). In free‐living mammals, the study of the HPA axis has relied primarily on blood and faecal samples (Palme et al. 2005; Palme 2019). While blood samples offer certain advantages, faecal samples allow for non‐invasive collection and provide an integrated measure of GC metabolites as these are pooled in faeces for a few hours and excreted with a time delay that roughly corresponds to an individual's gut passage time (Palme 2019). Importantly, faeces sampling does not induce the sudden increase in cortisol levels in blood that can occur due to handling stress during blood collection (Bosson, Palme, and Boonstra 2009). Faecal cortisol metabolites (FCMs) can serve as reliable indicators of stress, representing the cumulative unbound fraction of GCs over some time (usually a few hours, but depending upon the species; Palme et al. 2005). Nevertheless, steroid quantification using faecal samples also presents challenges. Faeces contain virtually no pure cortisol or corticosterone, but a mix of different metabolites. Composition and excretion of these metabolites vary between species and taxonomic relatedness offers limited predictive value (Touma and Palme 2005; Heistermann, Palme, and Ganswindt 2006). Thus, it is crucial to validate EIAs for each new species studied to ensure that the assay tracks biologically relevant changes in FCM levels, and consistently respond to the stressors encountered by the animal (Palme et al. 2005; Touma and Palme 2005; Palme 2019). Additionally, validation can reveal whether FCM values vary systematically across individuals, sexes, or age classes, which must be considered during interpretation (Markó et al. 2013). Despite results of unvalidated assays are unreliable, their use remains widespread (e.g., see Britnell et al. 2024).

We performed a biological validation experiment to test a set of group‐specific EIAs, an 11‐oxoaetiocholanolone (‘72T’; Möstl et al. 2002), a 5α‐pregnane‐3β,11β,21‐triol‐20‐one (‘37e’; Touma et al. 2003) and a tetrahydrocorticosterone (‘50c’; Quillfeldt and Möstl 2003) EIA, to measure FCMs in free‐living Alpine marmots *Marmot marmota*. Alpine marmots are large (2–6 kg), ground‐dwelling rodents inhabiting high Alpine and subalpine meadows in Europe. This species encounters various potential (anthropogenic) stressors, such as climate change or human disturbance through outdoor recreational activities in its habitat (Tafani et al. 2013; Ingold et al. 1993). Thus, there is an interest in assessing adrenocortical activity, and potential stress, in this species, possibly using non‐invasive methods, to understand ecological processes and physiological responses to anthropogenic disturbances. We used capture and handling during a relocation project as a stressful event to perform a biological validation experiment, to identify an EIA that can accurately assess biomarkers of endogenous stress generated by capture (Dickens, Delehanty, and Romero 2010; Palme 2019). We expect that, if reliable, an EIA would show an increase in FCMs around 10–24 h after the stress event, which roughly corresponds to the approximate gut passage time in most mammals (Palme 2019).

## 2 Materials and Methods

### 2.1 Data Collection

We studied marmots in the area of Livigno, in the Central Italian Alps (46°54′ N, 10°13′ E). The area is located at ~1800 m above the sea level and is characterised by a typical alpine climate with harsh winters and prolonged snow cover, and mild summers, with average yearly temperatures of −2°C (https://en.climate‐data.org/europe/italy/lombardy/livigno‐111597/). Marmots in the present study inhabited human‐managed alpine and subalpine pastures around Livigno. Their burrowing activities have caused conflicts with residents by degrading pastures, hindering mechanical mowing, and lowering fodder quality. In response, the municipality of Livigno initiated a relocation project (2021–2023) to reduce the species' impact on human activities. The translocation plan was planned for a duration of 3 years with a number of animals close to 500 (200, 150, 150 over different years). The plan was based on preliminary monitoring using line distance sampling (Corlatti et al. 2017) and was authorised by the Province of Sondrio in accordance with ISPRA (The Institute for Environmental Protection and Research). We used captures and handling from this relocation project as a biological validation experiment. Because capture, transportation and handling by humans are known to considerably increase HPA axis activity in various wildlife species, this is a well‐established method to validate GC assays in animals (Dickens, Delehanty, and Romero 2010; Palme 2019). An assay can be considered reliable if it can detect a significant increase in FCM concentration peaking approximately a few up to 24 h after the stress event (depending on individual gut retention time), followed by a subsequent decline (Palme 2019). When comparing different EIAs, the one that can detect the highest increase in FCM levels in response to a stressful event can be considered as most sensitive (Britnell et al. 2024; Palme 2019).

A total of 17 marmots (6 females and 11 males; 13 adults and 4 subadults) were included in the experiment (Table 1). No yearling marmots were subjected to the study. Marmots were captured with one‐door Tomahawk life‐traps, that were baited with dandelion *Taraxacum officinalis* and strategically placed close to burrow entrances. Traps were continuously monitored from a distant viewing point using binoculars or a spotting scope. Upon capturing an animal, two people calmly approached the cage, covered it with a blanket, and transferred the marmot into a sack. While trapped in a cage, marmots typically defecated (FZ, personal observation). These faeces were collected to determine individual baseline levels prior to the stress event. This is based on the knowledge that FCMs show up in excrements after delay that roughly matches the species‐specific gut transition time (Palme et al. 1996). The marmots were then transported to a checkpoint where health status was checked, physiological measurements (e.g., body length and body mass) were taken, and sex was determined by anogenital distance (Zelenka 1965). Age class (adult, or subadult) was determined through inspection of body length and mass. Additionally, animals were marked with a uniquely numbered plastic ear tag. Total handling time never exceeded 25 min. After handling, animals were transported in wooden boxes by car to the housing facilities (drive duration of approximately 30 min). Upon arrival, they were housed individually in opaque wooden boxes to reduce visual stimulation and help them calm down. In the facilities, human activities were kept to a minimum to avoid disturbances during the sample collection period. Marmot housing boxes had wire mesh bottoms to allow air circulation and to let excrement fall through for easy, non‐invasive collection without disturbing the animals. Fresh dandelion was provided *ad libitum* to meet their water and nutrient needs. The entire procedure was under the supervision of a veterinarian. Marmots were kept in the facilities until removal for relocation, which happened a few days after the current study was completed. We collected faecal samples at least every 4 h, both day and night, over a period of at least 48 h from capture. Because marmots defecated irregularly, at each sampling point, samples were only available for a subset of subjects. All collected samples were immediately stored at −18°C, and, after the end of the validation experiment, transferred to −24°C storage, where they were kept until laboratory analysis. For analysis, the samples were transported on dry ice to the lab for Experimental Endocrinology at the University of Veterinary Medicine Vienna (Austria). The samples were analysed in the same laboratory facilities over the course of 2 years, 2022 and 2023.

**TABLE 1:** Animals involved in the study and retained in the final dataset used for analysis, including information on sex (male/female), age class (adult/subadult), number of samples collected and time intervals of samples since capture (in hours).

Animal ID	Sex	Age class	Number of samples	Time sample since capture (in hours)	Retained in final dataset
209	Male	Subadult	4	0, 25, 29, 48	Yes
210	Male	Adult	5	1, 32, 46, 51, 55	Yes
211	Male	Adult	5	0, 17, 31, 41, 45	Yes
216	Male	Adult	7	0, 15, 21, 24, 28, 44, 48	Yes
217	Male	Adult	3	0, 19, 42	Yes
262	Male	Adult	4	0, 13, 34, 62	Yes
289	Male	Subadult	6	0, 18, 30, 52, 55, 90	Yes
379	Female	Subadult	9	0, 22, 31, 34, 56, 67, 74, 90, 96	Yes
380	Female	Adult	5	0, 27, 37, 48, 76	Yes
M5/205	Male	Adult	5	0, 8, 46, 72, 74	Yes
169	Female	Subadult	2	0, 29	No
170	Female	Adult	1	0	No
207	Male	Adult	3	0, 20, 58	No
286	Male	Adult	6	0, 49, 51, 53, 56, 93	No
290	Male	Adult	2	0, 67	No
350	Female	Adult	4	0, 23, 67, 69	No
351	Female	Adult	4	0, 27, 59, 63	No

### 2.2 Laboratory Analysis

FCMs were extracted by resolving a 0.5 g aliquot of sample in 5 mL of 80% methanol, while placed on a shaker, for 30 min. Subsequently, samples were centrifuged at 2500 *g* for 10 min at 8°C and the supernatants diluted 1:10 with assay buffer (Palme et al. 2013). FCMs were determined (in duplicate) with three different enzyme immunoassays (EIAs), screening for different groups of cortisol metabolites, to select the best suited EIA (Palme 2019). We utilised an 11‐oxoetiocholanolone (‘72T’; Möstl et al. 2002), a 5α‐pregnane‐3β,11β,21‐triol‐20‐one (‘37e’; Touma et al. 2003), and a tetrahydrocorticosterone (‘50c’; Quillfeldt and Möstl 2003) EIA. Details of the EIAs, including cross‐reactions with various FCMs can be found in the publications cited above. Intra‐ and inter‐assay coefficients of variation of all EIAs were below 10% and 15%, respectively. Sensitivity of the 72T, 37e and 50c EIA was 2.9, 1.8 and 2.2 ng/g faeces, respectively.

### 2.3 Statistical Analysis

To assess if and which EIA could map the event of capture, we used only animals for which at least 3 FCM values were available within 48 h from capture, to allow for the detection of non‐linear patterns, and avoid the occurrence of other stressors. Under the assumption that FCM values would show a delayed increase after capture, we used a modelling strategy that allows for a non‐linear variation in the response variable. Specifically, FCM values were modelled within an additive linear modelling framework, where FCM (response variable) was fitted as a non‐linear function of time since capture (explanatory variable, in hours), using the ‘gam’ function of the ‘mgcv’ package (Wood 2017). Sex and age class were added to the model as covariates to account for potentially different FCM baseline levels; prior to model fitting, the variance inflation factor (VIF) was inspected to assess collinearity. As FCM values are continuous positive data, which typically show a skewed distribution and non‐constant variance (see Corlatti 2018), we assumed a tweedie conditional distribution with log‐link function, which can accommodate both continuous and discrete data, and allows for free estimation of the index parameter to model the power‐variance relationship (Dunn and Smyth 2018). As the same individual was measured for at least 3 times, we fitted two models with different random structures, using restricted maximum likelihood (REML): one with time as random slope and animal ID as random intercept – which allows each individual to have a different temporal pattern of FCM variation, one with animal ID only as random intercept – which assumes all individuals have the same temporal pattern, but with different FCM baseline levels, and one with no random effects – which assumes there is no individual difference in temporal pattern and baseline FCM levels. Models were compared through the Akaike's Information Criterion corrected for small samples (AICc, Hurvich and Tsai 1989). Once the optimal random structure was found, the selected model was compared to a simpler model without the covariates sex and age class, using the AICc; both models were fitted using maximum likelihood (ML). Details are shown in Table 2. The top‐ranked model was then refitted using REML (Zuur et al. 2009) and inspected for residual distribution using the packages ‘mgcViz’ (Fasiolo et al. 2020) and ‘DHARMa’ (Hartig 2022). All analyses were conducted with R 4.3.1 (R Core Team 2023) in RStudio 2023.09.1 + 494 (Posit Team 2023) on both the 72T and the 37e EIAs. The 50c EIA was not analysed because it proved unreliable (i.e., detected FCM values were low compared to the other two EIAs and no peak was found).

**TABLE 2:** Models fitted to investigate variation in faecal cortisol metabolite data in marmots, obtained with different enzyme immunoassays (EIA). The table reports the used EIA, the selected structure in each step and whether models were fitted with restricted maximum likelihood (REML) or maximum likelihood (ML), degrees of freedom (df), the value of the Akaike's Information Criterion corrected for small samples (AICc), the delta AICc, the Akaike's weight (weight). In the model structure, ‘s(time)’ refers to the function used to define smooth terms, while ‘(time|ID)’ and ‘(1|ID)’ refers to random terms.

EIA	Selected structure	Model	df	AICc	ΔAICc	Weight
37e	Random (REML)	~ s(time) + sex + age class + (time|ID)	18	326.0	23.3	0.00
		~ s(time) + sex + age class + (1|ID)	13	302.7	0.0	1.00
		~ s(time) + sex + age class	6	384.0	81.3	0.00
	Fixed (ML)	~ s(time) + sex + age class + (1|ID)	12	302.5	0.1	0.49
		~ s(time) + (1|ID)	12	302.4	0.0	0.51
72T	Random (REML)	~ s(time) + sex + age class + (time|ID)	22	464.8	39.8	0.00
		~ s(time) + sex + age class + (1|ID)	15	425.0	0.0	1.00
		~ s(time) + sex + age class	6	453.0	28.0	0.00
	Fixed (ML)	~ s(time) + sex	14	424.1	0.1	0.49
		+ age class + (1|ID)	14	424.0	0.0	0.51
		~ s(time) + (1|ID)				

## 3 Results

**FIGURE 1:**
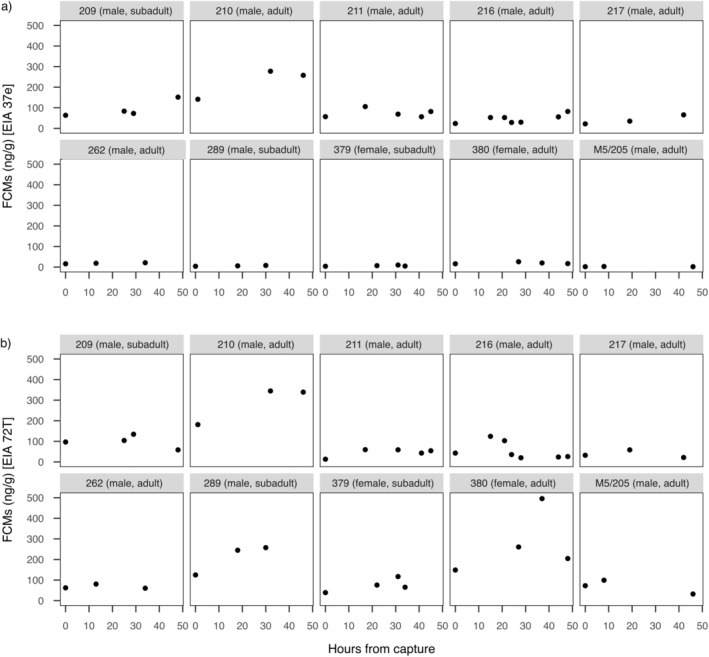
Raw faecal cortisol metabolite (FCM) values of individual (ID) Alpine marmots obtained with different enzyme immunoassays (EIA): (a) EIA 37e and (b) EIA 72T.

Only 10 individuals (8 males and 2 females; 7 adults and 3 subadults) were retained in the final dataset, which ensured to have enough data within 48 h from capture. Each individual showed different baseline levels of FCM, for both EIAs (Figure 1). With respect to the FCM temporal variation, the 72T EIA was more sensitive than the 37e EIA: the 37e EIA did not detect an increase in FCMs in five individuals (IDs 262, 289, 379, 380 and M5/205, Figure 1a) following the stress event, whereas the 72T EIA detected a change in FCM levels (Figure 1b).

**FIGURE 2:**
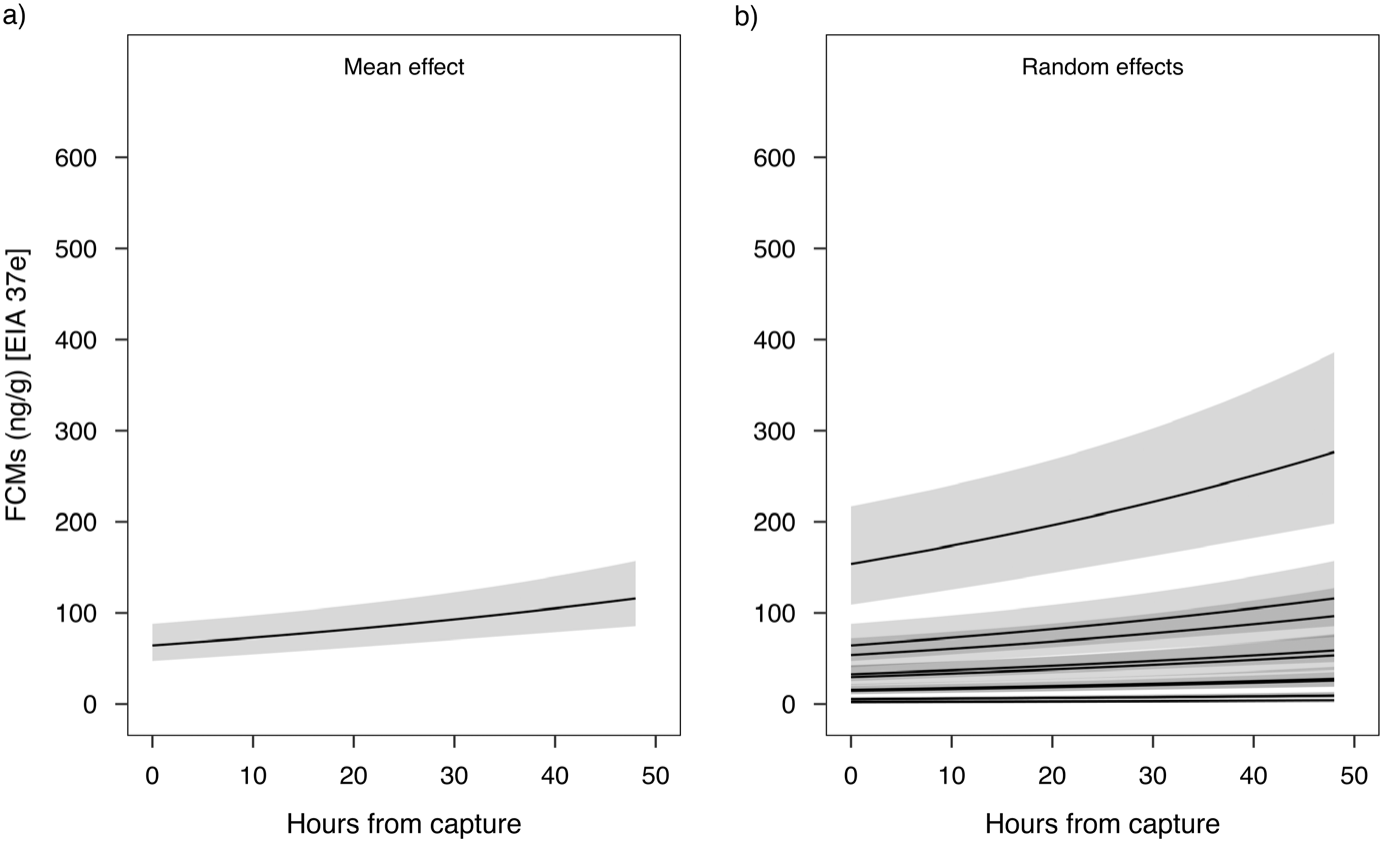
Predicted effects of hours from capture on levels of faecal cortisol metabolites (FCMs) of Alpine marmot with the 37e EIA: (a) mean effect and (b) individual effect. Grey shaded areas represent 95% confidence intervals.

**FIGURE 3:**
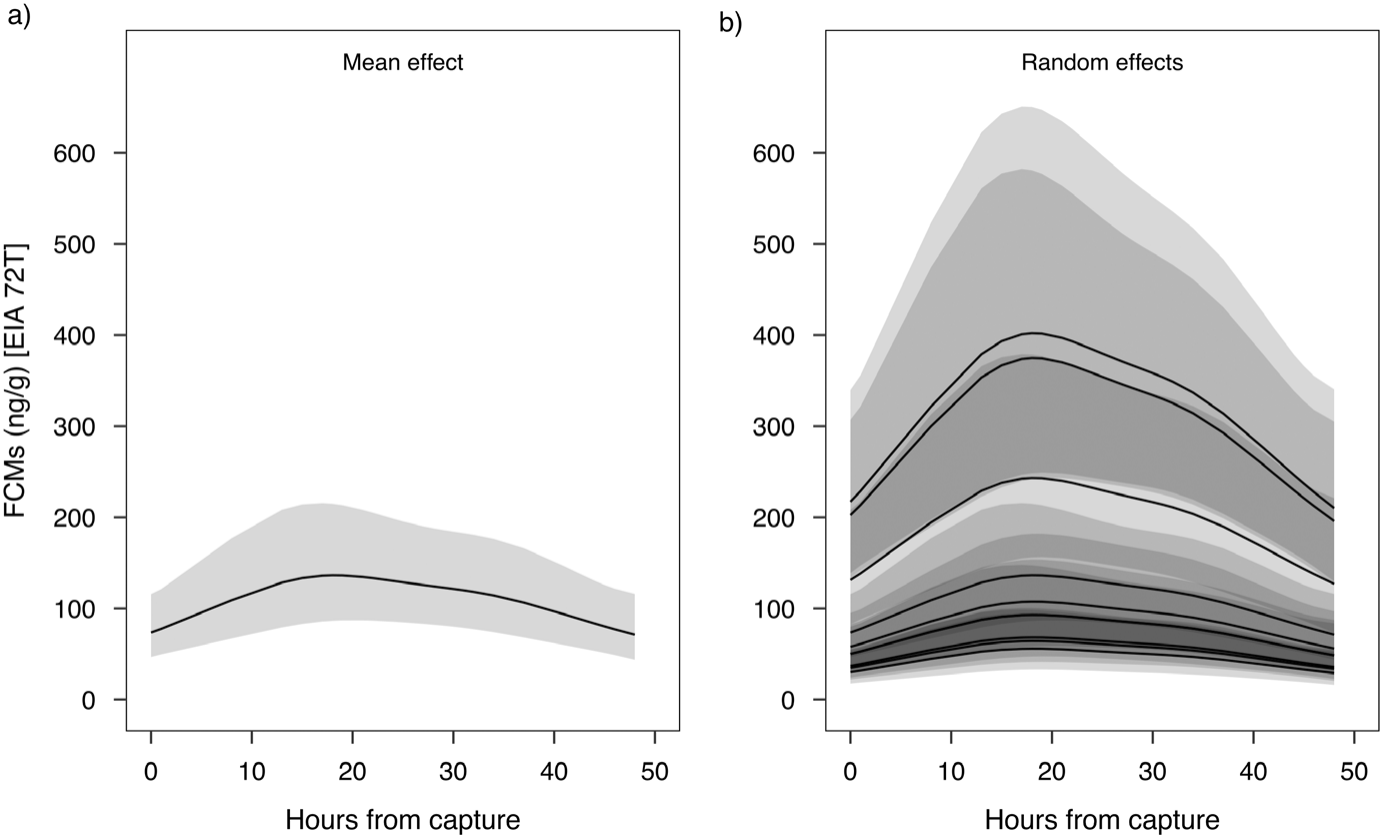
Predicted effects of hours from capture on levels of faecal cortisol metabolites (FCMs) of Alpine marmot with the 72T EIA: (a) mean effect and (b) individual effect. Grey shaded areas represent 95% confidence intervals.

The model selection procedure suggested that the best random structure was a random intercept with the individual as a random factor, for both EIAs; sex and age class were excluded as covariates from the final models for both EIAs (Table 2). Both models did not show any systematic pattern in the residuals, suggesting no major violations of assumptions. The EIA 37e model explained about 95% of the deviance, whereas the EIA 72T model explained about 84% of the deviance. The model for EIA 37e suggested a linear increase in the level of FCM with increasing time from capture (Figure 2a) and a marked individual effect (Figure 2b). The model for EIA 72T suggested a non‐linear effect of time from capture on FCM levels with a peak predicted at 18 h from capture (Figure 3a) and a marked individual effect (Figure 3b).

## 4 Discussion

We used a relocation project as a biological validation experiment to test the suitability of a set of EIAs to track variations in FCMs in Alpine marmots. Of the three assays tested, we identified one, the 72T EIA, that reliably detected changes in adrenocortical activity over time after a stressful event (i.e., capture). The 72T EIA detected an increase in FCM levels following the stressful event, peaking at 18 h, and then declining until 48 h post‐capture. In contrast, the 37e EIA was overall less sensitive than the 72T EIA and did not track an increase of FCM levels following the stressful event. Consequently, we cannot definitively identify which faecal steroids or metabolites are binding to the 37e EIA, nor can we confirm that they are indicative of HPA axis activity (Palme 2019). Both EIAs demonstrated inter‐individual variability in baseline FCM levels, while there was no evidence of an effect of sex or age class. Therefore, our results support the biological validity of the 72T EIA for assessing physiological stress through FCM levels in Alpine marmots and suggest that this EIA should be preferred over the 37e EIA in this species.

In our study species, predicted FCM levels peaked around 18 h, reflecting the approximate time‐lag between the stress event, secretion of GC hormones into the bloodstream, and the excretion of their metabolites via faeces (Palme 2019; Palme et al. 1996). This value falls within the expected window of approximately a few up to24 h for gut passage times in most mammals (Palme et al. 2005), and is roughly comparable to delay times in squirrels *Sciruidae* (e.g., European ground squirrels, 7.5 h [Strauss et al. 2007]; Columbian ground squirrels, 24 h [Bosson, Palme, and Boonstra 2009]; Yellow‐bellied marmots, 24 h [Smith et al. 2012]; chipmunks, 24 h [Hammond, Palme, and Lacey 2015]). Estimating the time delay between a stressful event and GC metabolites showing up in faeces, is crucial information derived from validations, as it is relevant to inform sampling interval in studies evaluating effects of acute stressors; not considering this time‐lag may result in missing FCM peak samples and thus, underrating or missing the impact of a stressor (Palme 2019). It should be noted that the predicted value of 18 h is approximate and is expected to vary individually, and/or related to activity, metabolic rate or diet for instance (e.g., Thorp, Ram, and Florant 1994; Palme et al. 1996, 2005; Goymann 2012).

Regulation of the HPA axis is typically highly individual, as well as metabolism and excretion of GCs (Palme 2005, 2019; Cockrem 2013). Thus, the observed individual difference in FCM baseline levels of Alpine marmots is not surprising. Individual differences may have different origins, such as genetics (Koolhaas et al. 1999), coping style (e.g., Pokharel and Brown 2023), or ecological experiences (e.g., Ros‐Simó and Valverde 2012; Mormède et al. 2007). Here, we assume that all subjects had experienced mostly similar environmental conditions as they came from the same area. However, differences in social environment such as social group structure, size or individual dominance rank, might have individually shaped FCM levels (Creel 2001; Hackländer, Möstl, and Arnold 2003). Additionally, we observed that two subjects had a relatively low increase in FCM levels following capture. It may be that capture might not have been experienced as stressful enough for these individuals to trigger a higher peak in FCMs (Fanson et al. 2017; Smith et al. 2012). In line, a previous study on behavioural effects of capture on Alpine marmots using similar capture protocol indicated that capture seemed at least not a highly traumatic experience (Giari et al. 2024). Likewise, research on yellow‐bellied marmots found that live‐trapping was only a stressor for some animals, particularly those without previous capture experience (Smith et al. 2012). Given that all tested animals came from managed pastures near human activities, it is possible that some individuals had a certain level of tolerance to humans (Samia et al. 2015).

In addition to individual differences, sex and age class (i.e., developmental stage) may influence GC and FCM levels. The underlying mechanisms may be physiological (e.g., adrenal activity influenced by sex‐specific steroid hormones [McCormick and Mathews 2007], or reproductive status, which is related to age [Harris and Saltzman 2013]), ecological (e.g., sex‐ or age‐specific social environments that affect perceived social stress [Creel et al. 2013]), or related to differences in GC metabolism (Raven and Taylor 1996; Kiess et al. 1995; Palme et al. 2005). Presently, we did not find evidence for an effect of sex or age class (adult or subadult) on FCM baseline values. However, because the majority of our test subjects were adult males, these results should be interpreted cautiously. There is no previous work on FCM in Alpine marmot, but there is some knowledge available on age and sex effects on GC and metabolite levels available from other matrices. In line with our findings, Costantini et al. (2012) found no evidence for sex differences in Alpine marmot blood plasma cortisol concentrations. On the contrary, the authors reported age‐related differences in plasma cortisol concentrations, with higher levels in subadult when compared with adult (and yearling) marmots. Another study investigating hair cortisol concentrations (HCC) in Alpine marmot reports higher HCC levels in females when compared with males but found no effect of age class (Zenth et al. 2022). Yet, it should be noted that results derived from different matrices may not be directly comparable. For example, hair gives a different picture than the other matrices as it integrates baseline GC levels and short‐term peaks over more long‐term periods (Sheriff et al. 2011), whereas different than faeces or hair, blood plasma GC concentration is a point‐in‐time measure, where many other factors could mask sex effects (Sheriff et al. 2011). Irrespective of the underlying mechanisms, considering potential confounders of FCMs is crucial to avoid potential biases. In wildlife research, it is often challenging to assign samples to a particular individual (i.e., which could be done either by observing a defecting animal [Ganswindt et al. 2010] or with genetic analysis [Rehnus and Palme 2017]). Here, previous knowledge derived from experimental setups or captive animals is vital to be aware of, or, in the best case, be able to a priori exclude, confounding effects. Our work paves the way for further studies using FCM levels as non‐invasive, feedback free indicators of (anthropogenically mediated) stress in this species. We encourage further studies with larger sample sizes, potentially including yearling animals, to investigate the effects of sex and age class on FCMs in the Alpine marmot.

## Author Contributions

**Friederike Zenth:** conceptualization (supporting), data curation (equal), investigation (equal), methodology (supporting), visualization (equal), writing – original draft (equal), writing – review and editing (equal). **Elena Morocutti:** data curation (equal), formal analysis (supporting), investigation (equal), writing – original draft (equal), writing – review and editing (equal). **Rupert Palme:** conceptualization (supporting), formal analysis (supporting), funding acquisition (equal), investigation (equal), methodology (equal), project administration (equal), supervision (equal), writing – original draft (equal), writing – review and editing (equal). **Sandro Nicoloso:** conceptualization (supporting), funding acquisition (equal), project administration (equal), resources (equal), writing – review and editing (equal). **Stefano Giacomelli:** data curation (equal), investigation (equal), writing – review and editing (equal). **Sabine Macho‐Maschler:** data curation (equal), investigation (equal), methodology (equal), supervision (equal), writing – review and editing (equal). **Ilse Storch:** funding acquisition (equal), supervision (equal), writing – review and editing (equal). **Luca Corlatti:** conceptualization (lead), data curation (equal), formal analysis (lead), funding acquisition (equal), investigation (supporting), methodology (lead), project administration (lead), resources (equal), supervision (lead), visualization (lead), writing – original draft (equal), writing – review and editing (equal).

## Ethics Statement

All procedures performed during captures were approved by the Province of Sondrio (PARERE N. 9 DEL 09/05/2022) in accordance with ISPRA (The Institute for Environmental Protection and Research) (Ref. Int. 19086/2021).

## Conflicts of Interest

The authors declare no conflicts of interest.

## Data Availability

Data are available from the Dryad Digital Repository: https://doi.org/10.5061/dryad.3r2280gsc
